# *Orthodenticle *is necessary for survival of a cluster of clonally related dopaminergic neurons in the *Drosophila *larval and adult brain

**DOI:** 10.1186/1749-8104-6-34

**Published:** 2011-10-14

**Authors:** Jorge Blanco, Rahul Pandey, Martin Wasser, Gerald Udolph

**Affiliations:** 1 Institute of Medical Biology, 8A Biomedical Grove, Singapore 138648; 2 Institute of Biotechnology, University of Helsinki, Viikinkaari 1, PO Box 65, Fin-00014 Finland; 3 Bioinformatics Institute, 30 Biopolis Street, #07-01 Matrix, Singapore 138671

## Abstract

**Background:**

The dopaminergic (DA) neurons present in the central brain of the *Drosophila *larva are spatially arranged in stereotyped groups that define clusters of bilaterally symmetrical neurons. These clusters have been classified according to anatomical criteria (position of the cell bodies within the cortex and/or projection pattern of the axonal tracts). However, information pertaining to the developmental biology, such as lineage relationship of clustered DA neurons and differential cell subtype-specific molecular markers and mechanisms of differentiation and/or survival, is currently not available.

**Results:**

Using MARCM and twin-spot MARCM techniques together with anti-tyrosine hydroxylase immunoreactivity, we have analyzed the larval central brain DA neurons from a developmental point of view and determined their time of birth, their maturation into a DA neurotransmitter phenotype as well as their lineage relationships. In addition, we have found that the homeodomain containing transcription factor Orthodenticle (Otd) is present in a cluster of clonally related DA neurons in both the larval and adult brain. Taking advantage of the *otd *hypomorphic mutation *ocelliless *(*oc*) and the *oc2-Gal4 *reporter line, we have studied the involvement of *orthodenticle *(*otd*) in the survival and/or cell fate specification of these post-mitotic neurons.

**Conclusions:**

Our findings provide evidence of the presence of seven neuroblast lineages responsible for the generation of the larval central brain DA neurons during embryogenesis. *otd *is expressed in a defined group of clonally related DA neurons from first instar larvae to adulthood, making it possible to establish an identity relationship between the larval DL2a and the adult PPL2 DA clusters. This poses *otd *as a lineage-specific and differential marker of a subset of clonally related DA neurons. Finally, we show that *otd *is required in those DA neurons for their survival.

## Background

The *Drosophila *adult brain is a highly organized and complex structure that contains thousands of neurons (in the order of 10^5^) [1] exhibiting multiple cell-type identities, as characterized by various morphological, electrophysiological and molecular features. All these neurons arise from the mitotic activity of a small number of progenitor cells (neuroblasts (NBs)), which generate lineages of clonally related neurons via two proliferative phases of neurogenesis [2,3]. The first phase of neurogenesis takes place during embryogenesis and starts with the specification and delamination of the NBs from the procephalic neurogenic region. Between embryonic stages 8 and late 11, around 100 NBs delaminate from this region on either side of the embryo in a reproducible spatiotemporal pattern [4]. Each NB assumes a unique identity, as revealed by the expression of a specific set of marker genes such as the proneural genes, gap genes and segment polarity genes [5-7], and gives rise to an invariant cell lineage through multiple rounds of asymmetric cell divisions. In each cell division, the NB self-renews and generates a smaller daughter cell (named the ganglion mother cell), which divides only once to give rise to two post-mitotic neurons or glial cells (reviewed in [8-10]). The first neurogenic process terminates at the end of embryogenesis, when most NBs stop dividing and enter a dormant phase called quiescence [11]. The cells so far generated (primary neurons) wire the larval nervous system and eventually may remodel during metamorphosis to contribute to the adult brain [12,13]. The second phase of neurogenesis starts during the late first (L1) and second (L2) instar larval stage, when the inactive NBs resume mitotic activity and, through rounds of asymmetric cell divisions, generate the population of secondary neurons and glial cells that accounts for more than 90% of the adult brain [11,14]. Hence, a complete NB lineage can be divided into two discrete cell populations, each containing the cells generated by the NB during distinct developmental phases (primary neurons during embryogenesis and secondary neurons during larval development) and each harboring multiple neuronal cell types. It has been proposed that neuron identity within a NB lineage depends on a combination of spatial and temporal cues provided, firstly, by the unique identity the NB acquires during its specification/delamination time [7,15] and, secondly, as a result of a birth time/order-dependent mechanism [16], whereby cell-type specification of the nascent post-mitotic neurons depends on the identity of the progenitor temporal transcription factor expressed by the NB at each particular time during lineage progression [17].

The homeobox gene *orthodenticle *(*otd*), as a cephalic gap gene, is expressed in broad domains of the procephalic ectoderm during early neurogenesis, covering most of the protocerebral anlage and the anterior part of the deutocerebral anlage [18]. Subsequently, its expression is also detected in NBs delaminating from these domains, where it plays instructive roles important for cell viability and spatial identity of the nascent NBs [5]. It has also been proposed that *otd *might control brain NB formation by triggering proneural gene expression [19]. Inactivation of *otd *at this early embryonic phase impairs NB formation and leads to a gap-like phenotype in the anterior head that includes the deletion of the protocerebral anlage and part of the deutocerebral anlage [20]. Later in development, *otd *expression is also detected in post-mitotic neurons of the developing brain and ventral nerve cord, not only in the embryo, but also in the larval and pupal brain and even in mature neurons of the adult brain. In this regard, flies homozygous for an *otd *viable hypomorphic mutation called *ocelliless *(*oc*) show developmental defects that affect the protocerebral bridge, an important neuropile structure in the adult brain that is part of the central complex [20]. However, whether this phenotype is due to the altered expression of *otd *in progenitor cells, post-mitotic cells or both is not known.

A comprehensive study of gene function during neuronal cell fate specification requires a previous and thorough cell lineage analysis and demands cell type-specific molecular markers to trace the cells under study. In this paper, we have focused our attention on the array of dopaminergic (DA) neurons that populate the *Drosophila *central brain during larval development. We have used cell lineage tracing genetic techniques together with immunoreactivity against the enzyme tyrosine hydroxylase (TH), the rate-limiting enzyme in dopamine biosynthesis [21,22], to study their development, phenotypic maturation and lineage relationships. Interestingly, one cluster of clonally related DA neurons expresses *otd *from early L1 to adulthood, allowing us to examine the post-mitotic role of *otd *in controlling identity and/or survival of DA neurons in the *Drosophila *larval and adult brain.

## Results

### Birth, clustering and phenotypic maturation of dopaminergic neurons in the larval central brain

**Figure 1:**
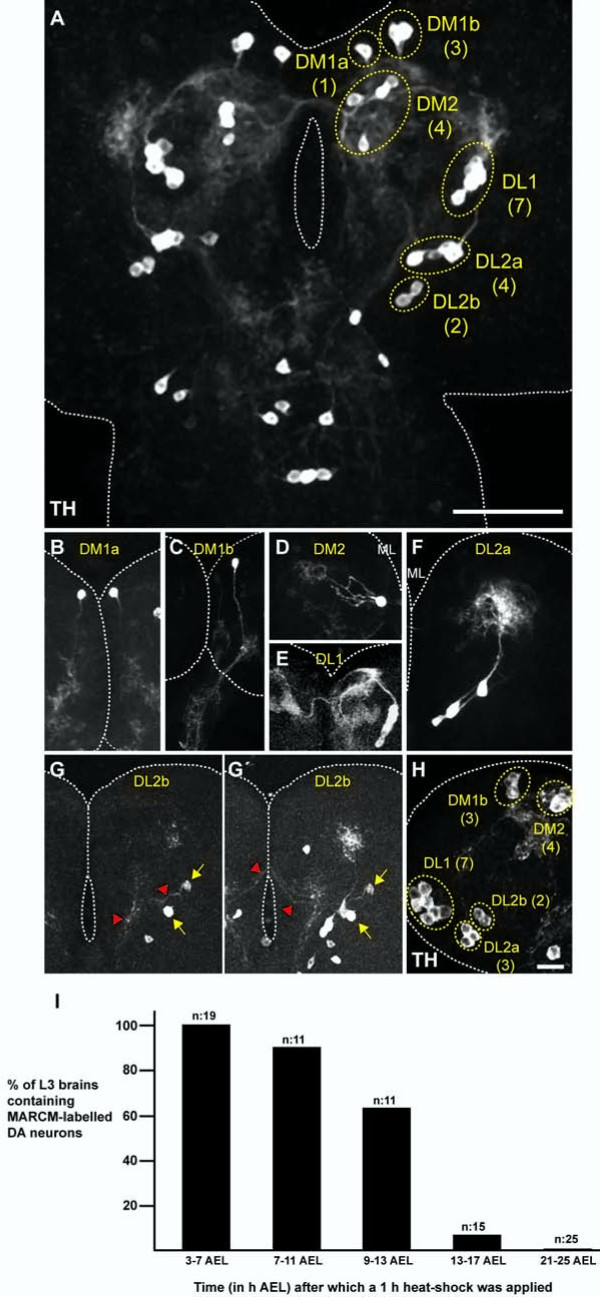
**Birth, clustering and differentiation of larval central brain dopaminergic neurons**. **(A) **Third instar larval stage (L3) brain showing bilaterally symmetrical groups of TH-positive DA neurons clustered according to the position of the cell bodies within the cortex (the number of neurons per cluster is given in parentheses) and the neurite projection patterns. Scale bar: 50 μm. **(B-G') **Neurite projection patterns. Wild-type cell clones were induced in progenitor cells during early embryogenesis (3 to 7 h after egg laying (AEL)) and analyzed during L3. The clones were labeled with membrane tethered green and red flourescent proteins (both in grey) using the Twin-spot MARCM technique together with the *TH-gal4 *driver. When possible, neurite projection patterns of the entire cell cluster are shown (B,E,F); otherwise, just one DA neuron as a representative for the cell cluster is produced (C,D). (G,G') The neurite projection pattern (red arrowheads) of DL2b DA neurons (yellow arrows) is shown in two consecutive confocal optical sections. **(H) **Early L1 brain (24 to 28 h AEL) showing clusters of TH-positive cells (the number of DA neurons per cluster is given in parentheses) Scale bar: 10 μm. **(I) **Most larval DA neurons in the central brain are born during early neurogenesis between 3 to 13 h AEL. Wild-type cell clones, labeled with the MARCM technique in combination with the *TH-gal4 *driver, were induced in progenitor cells at different times during development as indicated and analyzed in L3 brains. Images, except for (G,G'), represent Z projections of individual confocal optical sections. AEL, after egg laying; DA, dopaminergic; DL, dorso lateral; DM, dorso medial; L, instar larval stage; MARCM, mosaic analysis with a repressible cell marker; ML, midline; TH, tyrosine hydroxylase.

Using immunoreactivity against the enzyme TH as a DA neuron-specific molecular marker, an array of 21 DA neurons could be visualized in the central part of each brain hemisphere during the third instar larval stage (L3; Figure 1A). The stereotypical arrangement of their cell bodies in various groups has been used previously to define four clusters of DA neurons that occupy distinct anatomical positions in the central brain [23-25]: dorso medial 1 cluster (DM1: four cells), dorso medial 2 cluster (DM2: four cells), dorso lateral 1 cluster (DL1: seven cells) and dorso lateral 2 cluster (DL2: six cells). As judged by their projection and innervation patterns, DM2 and DL1 cell clusters contained apparently homogeneous populations of DA neurons. On the contrary, both DM1 and DL2 clusters could be further subdivided into two subclusters according to their differential projection patterns. A single neuron in the DM1 cluster (named DM1a) projected ventrally into the lower part of the ipsilateral brain lobe (Figure 1B), whereas the remaining three DM1 DA neurons (named the DM1b cell cluster) innervated more ventrally localized regions of the brain lobe (subesophagial ganglion) and further extended into the thoracic segments of the ventral ganglion (Figure 1C). DM2 neurons projected ipsilaterally into the anterior part of the protocerebrum (Figure 1D), whereas DL1 neurons were characterized by dorsally projecting neurites that bifurcated into dorsal and ventral branches before crossing the midline to reach the contralateral brain lobe (Figure 1E). Although described as homogeneous, DM2 and DL1 cell clusters are possibly heterogeneous at the single-cell level. Indeed, six different cell types with slightly different innervation patterns have been described within the DL1 cell cluster using single-cell labeling techniques [25]. Similarly to the DM1cell cluster, the six DL2 DA neurons displayed two distinct projection patterns: four neurons (named the DL2a cell cluster) projected their neurites dorsally into the anterior part of the ipsilateral brain lobe (Figure 1F). The remaining two cells (named the DL2b cell cluster) projected laterally and arborized in the ventral part of the brain lobe before crossing the midline to innervate the contralateral brain lobe (Figure 1G,G').

We next determined the time of birth of the larval central brain DA neurons by using the MARCM (mosaic analysis with a repressible cell marker) technique in combination with the *TH-Gal4 *driver. Flippase recognition target (FRT)-mediated mitotic recombination was randomly induced, exposing progenitor cells to a one hour heat-shock treatment (37°C) at different developmental stages and L3 brains were assayed for the presence of green fluorescent protein (GFP)-labeled DA neurons. When mitotic recombination was induced during early L1, a time point commonly used to label neurons born during larval development, GFP-positive DA neurons were not detected in the resulting wild-type cell clones. This result agreed with the observation that *TH *expression was already detectable in early L1 brains (Figure 1H). On the contrary, a heat-shock treatment applied during early embryogenesis efficiently labeled DA neurons in the L3 central brain, with the highest labeling efficiencies achieved when cell clones were induced at early embryonic stages (between 3 and 7 and 7 and 11 hours after egg laying (AEL); Figure 1I). These results demonstrate that DA neurons present in the L3 central brain are primary neurons that arise during early embryogenesis.

Despite their early embryonic origin, the larval central brain DA neurons did not express the cell type-specific marker gene *TH *during embryogenesis, and even at late embryonic stages (stage 17) anti-TH immunoreactivity in the central nervous system was restricted to the ventral nerve cord (data not shown and [24]). However, during early L1 (24 to 28 h AEL) most of the central brain DA neurons already displayed *TH *expression (Figure 1H), with two exceptions: the DM1a DA neuron started to show anti-TH labeling during mid-late L1, whereas a DL2a DA neuron only showed anti-TH immunoreactivity at mid-late L3 (data not shown).

In summary, DA neurons present in the central brain of the *Drosophila *larva at L3 are generated during early embryogenesis and most of them acquire a mature neurotransmitter phenotype during early L1.

### Dopaminergic neurons present in the central brain during larval development are generated by seven neuroblast lineages

In *Drosophila*, clonally related neurons typically remain clustered in the mature brain and project their neurites into specific neuropile compartments [26,27]. To address whether the anatomical clustering of DA neurons in the L3 central brain is due to a clonal origin, we analyzed lineage relationships among DA neurons in each cell cluster. For this purpose, we utilized the twin-spot MARCM technique in combination with the *tubulin-Gal4 *driver. Wild-type cell clones were induced during early embryogenesis (3 to 7 h AEL) and assayed in early L1 brains (24 to 28 h AEL) for the presence of GFP- and red fluorescent protein (RFP)-labeled NB clones containing TH-positive neurons. Although at this developmental stage two DA neurons still did not express the cell type-specific marker gene *TH *and thus could not be considered in our analysis, two reasons justified our decision. Firstly, the reduced number of total cells present in the larval brain during L1 facilitated an easier lineage analysis. Secondly, we observed that the *tubulin *promoter underwent partial down-regulation in primary neurons during L3 (data not shown), impairing a reliable analysis of the lineage relationships among DA neurons.

**Figure 2:**
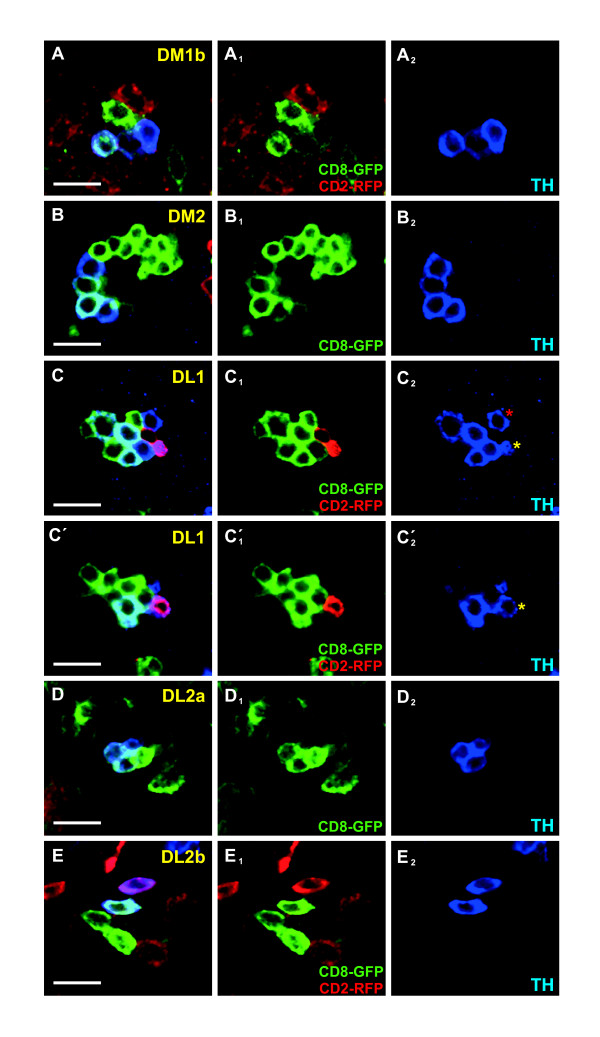
**Twin-spot MARCM lineage analysis of larval central brain dopaminergic neurons at L1 reveals clonal relationship between dopaminergic neurons**. **(A-E) **Membrane-tethered GFP- and RFP-labeled wild-type NB clones containing TH-positive (blue) DA neurons from different DA cell clusters: DM1b cluster (A), DM2 cluster (B), DL1 cluster (C,C'), DL2a cluster (D) and DL2b cluster (E). All panels represent individual confocal optical sections (0.5 μm thick). The red asterisk in C_2 _identifies the DL1 DA neuron that belongs to a different NB lineage (DL1b). The yellow asterisk in C_2 _and C'_2 _identifies the same neuron in different optical sections. Scale bars: 10 μm. DA, dopaminergic; DL, dorso lateral; DM, dorso medial; GFP, green fluorescent protein; L, instar larval stage; NB, neuroblast; RFP, red fluorescent protein; TH, tyrosine hydroxylase.

Our analysis revealed that all the DA neurons assigned to the DM1b (Figure 2A), DM2 (Figure 2B), DL2a (Figure 2D) and DL2b (Figure 2E) clusters were contained within individual NB clones and thus were clonally related. By contrast, we only found NB clones containing at most six out of the seven DL1 DA neurons (Figure 2C,C'), indicating that two NBs generate the seven DL1 DA neurons.

As mentioned above, due to their delayed *TH *expression, two DA neurons were initially not included in the lineage analysis. In order to validate their allocation to the respective DA cell clusters, we induced MARCM-labeled wild-type NB clones during early embryogenesis (3 to 7 h AEL) and analyzed L3 brains. We tried to circumvent the down-regulation of the *tubulin *promoter by including an additional copy of the *UAS-CD8::GFP *transgene in the genotype of the analyzed larvae. Although this strategy did not always work reliably (for example, we did not observe single NB clones containing the group of six DL1 DA neurons), we were still able to assign the missing DM1 DA neuron to an independent NB lineage (named DM1a), as well as to confirm the allocation of the missing DL2 DA neuron to the DL2a NB lineage (Additional file 1).

In summary, we found that seven NB lineages generate the DA neurons present in each hemisphere of the *Drosophila *larval central brain and that their clustered appearance is, at least in part, a consequence of a clonal relationship. Moreover, the assignment of DA neurons to particular NB lineages provides access to study genetic mechanisms of DA neuron cell fate specification.

### *orthodenticle *is expressed in DL2a dopaminergic neurons

**Figure 3:**
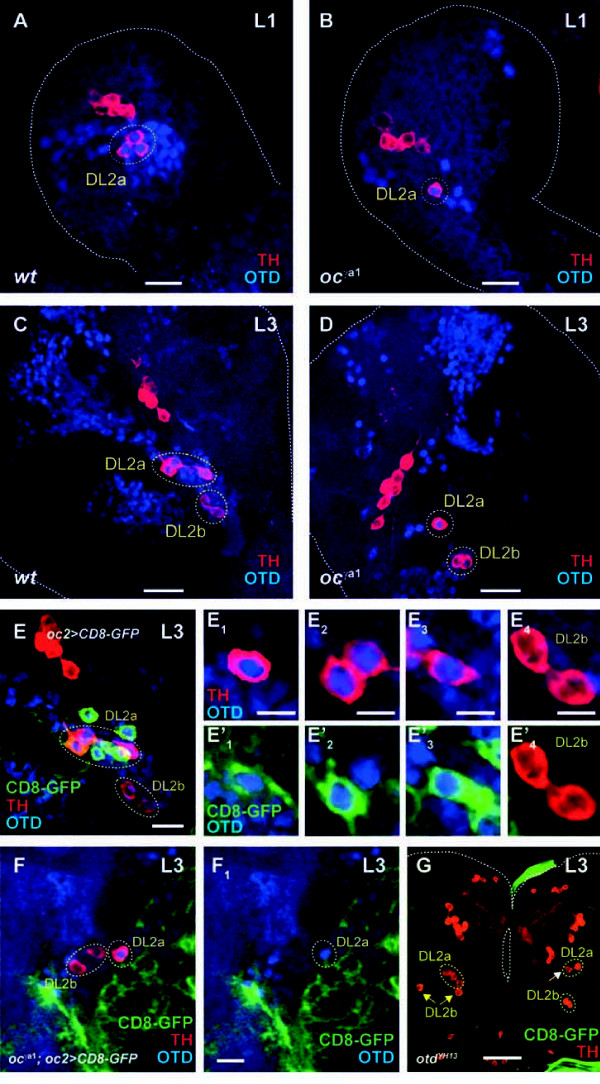
***otd *is expressed in DL2a dopaminergic neurons in the larval brain**. **(A,C) ***otd *(blue) is expressed in a specific cluster of TH-positive (red) DA neurons of the larval central brain during L1 (A) and L3 (C). **(B,D) **In *oc*^*γa1 *^hemizygous larvae, two out of three DL2a DA neurons at L1 (B) and three out of four DL2a DA neurons at L3 (D) are not detected using anti-TH immunoreactivity. Scale bars: 10 μm (A,B); 20 μm (C,D). **(E) **The *oc2 *enhancer is active in three out of the four DL2a DA neurons at L3. Scale bar: 10 μm. **(E**_**1**_**-E**_**3**_**) ***otd *expression and **(E'**_**1**_**-E'**_**3**_**) ***oc2 *enhancer activity in DL2a DA neurons are analyzed in individual optical sections. **(E**_**4**_**,E'**_**4**_**) **DL2b DA neurons display neither *otd *expression nor *oc2 *enhancer activity. Scale bars: 5 μm (E_1_-E_4_). **(F,F**_**1**_**) **In L3 *oc*^*γa1 *^hemizygous larvae, *oc2 *enhancer activity in DL2a DA neurons is lost. Scale bar: 10 μm. **(G) ***otd*^*YH13 *^mutant cell clones induced during early embryogenesis impair the development of DL2a DA neurons (white arrow). Yellow arrows point to the DL2b DA neurons in the left hemisphere Scale bar: 50 μm. All the panels, except for (E_1_-E_4_) and (E'_1_-E'_4_), represent Z projections of individual confocal optical sections. DA, dopaminergic; DL, dorso lateral; GFP, green fluorescent protein; L, instar larval stage; *oc*, *ocelliless*; *otd*, *orthodenticle*; TH, tyrosine hydroxylase.

The homeobox containing gene *otd *is broadly expressed in the anterior ectoderm during the blastoderm stage and is necessary for the specification of most of the NBs that populate the protocerebrum and part of the deutocerebrum [5,19]. Using Otd immunostainings, we found that *otd *was expressed in DL2a DA neurons already at early L1 (Figure 3A). At mid-late L3, when the entire complement of DA neurons in the larval central brain was already visible with anti-TH labeling, Otd was specifically detected in the four DL2a DA neurons (Figure 3C,E_1_-E_3_).

To analyze the significance of *otd *expression in the specification and/or survival of post-mitotic DL2a DA neurons, we made use of the hypomorphic *otd *allele *oc*. *oc *hemizygous flies are viable, but they show a lack of ocelli and associated bristles in the head vertex. These flies also lack the protocerebral bridge, a neuropile structure present in the fly adult brain that is part of the central complex [20]. These phenotypes arise as a consequence of chromosomal rearrangements that remove *cis*-acting regulatory sequences (*oc *enhancer) important for *otd *expression during ocelli and protocerebral bridge development [18,28]. During early L1, *otd *expression in *oc *hemizygous larvae was downregulated and the number of TH-positive DL2a DA neurons was reduced to one or two neurons, as compared to the three DL2a DA neurons present in wild-type brains (Figure 3B). In L3 brains, the lack of anti-TH immunoreactivity affected three out of the four DL2a DA neurons (Figure 3D). To investigate whether this phenotype is due to the loss of DL2a DA neurons *per se *or to *TH *downregulation in these neurons, we made use of the *oc *enhancer and the *oc2-Gal4 *driver [28] as an alternative way of labeling DL2a DA neurons. Detection of *oc *enhancer activity in DL2a DA neurons in *oc *mutant L3 brains would imply that *otd *is involved in the activation and/or maintenance of *TH *expression in these neurons. By contrast, the absence of *oc *enhancer activity in DL2a DA neurons in *oc *mutant L3 brains would indicate *otd *is primarily necessary for survival of DL2a DA neurons. Reporter gene expression under the control of the *oc2-Gal4 *driver was detected in three out of the four DL2a DA neurons in wild-type L3 brains (Figure 3E'_1_-E'_3_). Interestingly, when *oc2-gal4 *transcriptional activity was analyzed in *oc *mutant L3 brains, reporter gene expression was not detected in DL2a DA neurons (Figure 3F,F_1_).

We also analyzed the relevance of *otd *expression during embryogenesis for the generation of DL2a DA neurons. We induced MARCM-labeled cell clones mutant for a null *otd *allele during early embryogenesis and assayed L3 brains for the presence of TH-positive cells within these *otd *mutant clones. Very few *otd*- NB clones were recovered in the central brain and none of them affected the DL2a DA neurons. However, L3 brains lacking some of the DL2a DA neurons were observed (white arrow in Figure 3G), though the corresponding NB clone was not detected. The simplest interpretation for this result is that Otd depletion during early neurogenesis induces NB cell death and, as a result, loss of DL2a DA neurons.

Taken together, the lack of anti-TH labeling observed in three DL2a DA neurons in *oc *mutant L3 brains is not due to *TH *downregulation, but likely reflects the absence of these neurons. Thus, we conclude that *otd *is necessary for survival of the larval DL2a DA neurons.

### Adult PPL2 dopaminergic neurons derive from the larval DL2a cluster

**Figure 4:**
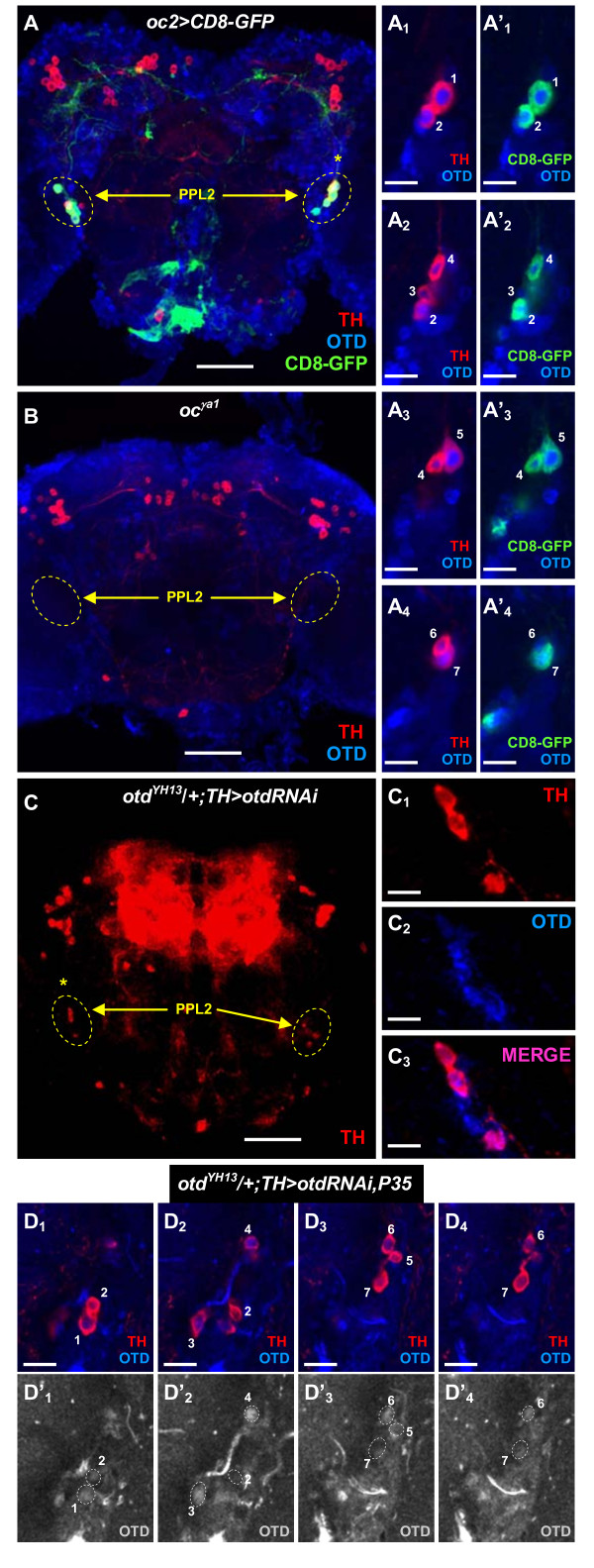
***otd *is expressed in PPL2 dopaminergic neurons in the adult brain**. **(A) ***otd *(blue) expression in the PPL2 cell cluster of TH-positive (red) DA neurons in the adult brain. The PPL2 cell cluster labeled with a yellow asterisk is magnified and *otd *expression **(A**_**1**_**-A**_**4**_**) **and *oc2 *enhancer activity **(A'**_**1**_**-A'**_**4**_**) **are analyzed in single optical sections. Scale bars: 10 μm. **(B) **In *oc*^*γa1 *^hemizygous flies, the seven PPL2 DA neurons are not detected using anti-TH immunoreactivity. **(C) **Targeted expression of an *otd*-specific RNA interference construct in DA neurons (using the *TH-gal4 *driver) impairs the viability of four PPL2 DA neurons in the adult brain of *otd*^*YH13 *^heterozygous flies (7 days old). **(C,C**_**1**_**-C**_**3**_**) ***TH *and *otd *expression in the resulting PPL2 cluster (labeled with a yellow asterisk in (C)) is analyzed at higher magnification. Scale bars: 10 μm. **(D**_**1**_**-D**_**4**_**) **Targeted expression of *P35 *in Otd-depleted DA neurons (using the *TH-gal4 *driver) rescues cell viability and *TH *expression in PPL2 DA neurons in the adult brain of *otd*^*YH13 *^heterozygous flies (7 days old). Pictures represent single optical sections. Scale bars: 10 μm. **(D'**_**1**_**-D'**_**4**_**) **Otd protein levels in four PPL2 DA neurons (1, 2, 5 and 7) are not recovered. Panels (A-C,C_1_-C_3_) represent Z projections of individual confocal optical sections. Scale bars in (A-C): 50 μm. DA, dopaminergic; GFP, green fluorescent protein; *oc*, *ocelliless*; *otd*, *orthodenticle*; PPL, protocerebral posterior lateral; RNAi, RNA interference; TH, tyrosine hydroxylase.

Similarly to the larval brain, DA neurons also cluster in the adult *Drosophila *brain and these clusters have been annotated according to their anatomical position [23,24,29]. In order to investigate whether adult DA neurons express *otd*, we assayed wild-type young adult brains (3 to 7 days old after eclosion) for the co-expression of *otd *and *TH*. We observed that seven DA neurons, assigned to the protocerebral posterior lateral 2 (PPL2) cluster, expressed *otd *(Figure 4A,A_1_-A_4_) and five of them also showed *oc2 *enhancer transcriptional activity (Figure 4A,A'_1_-A'_4_). Interestingly, the entire PPL2 cluster was not detected in *oc *mutant hemizygous flies using anti-TH immunoreactivity (Figure 4B). Also, targeted knockdown of *otd *in DA neurons by RNA interference (RNAi) resulted in a partial phenocopy of this mutant phenotype (*TH *expression was detected in three out of the seven PPL2 DA neurons; Figure 4C,C_1_-C_3_) and this effect could be rescued by the simultaneous expression of the anti-apoptotic gene *P35 *(Figure 4D_1_-D_4_). Otd protein levels in the rescued DA neurons (cells 1, 2, 5 and 7) were indistinguishable from background levels or drastically reduced (Figure 4D'_1_-D'_4_); yet, cell viability and *TH *expression were recovered by *P35 *co-expression. These results indicate that *otd *is dispensable for *TH *expression in PPL2 DA neurons and it is mainly required for their survival in the adult brain.

**Figure 5:**
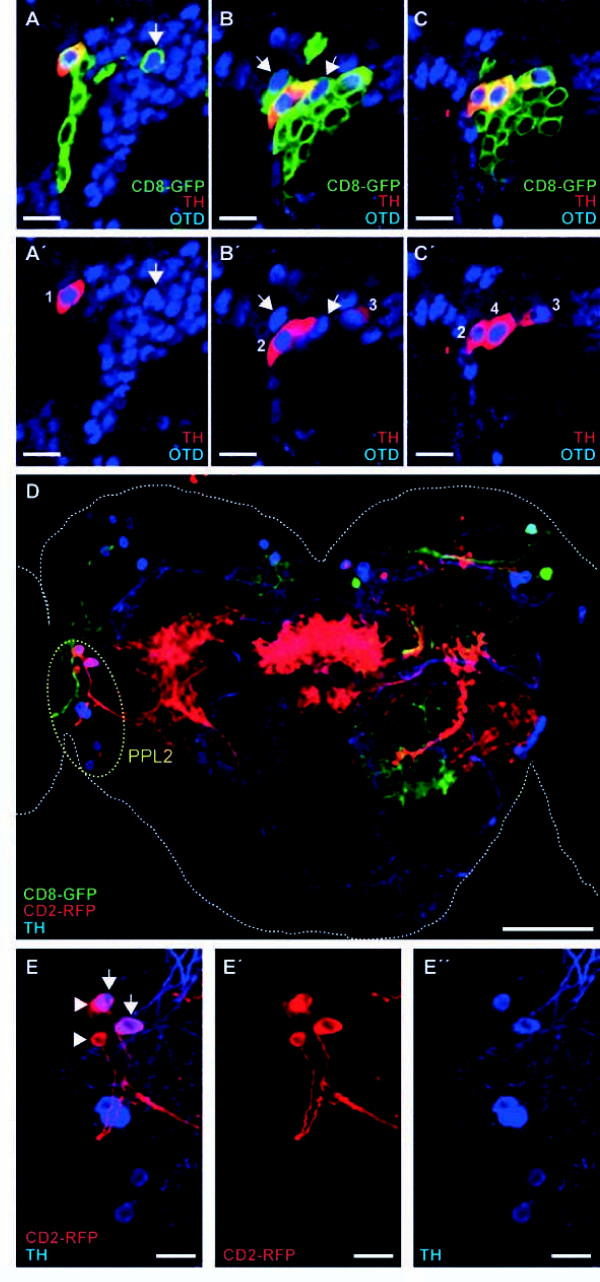
**PPL2 dopaminergic neurons in the adult brain are lineage related to the larval DL2a dopaminergic cell cluster**. **(A-C') **DL2a NB lineage (labeled with membrane tethered GFP) was analyzed during L3 using individual optical sections. Within the NB clone, seven cells are Otd-positive (blue). Three of them (arrows) only express *otd*, whereas the other four express both *otd *and *TH *(red). Scale bars: 10 μm. **(D) **Twin-spot MARCM-labeled wild-type cell clones were induced during early L1 and analyzed in the adult brain. Scale bar: 50 μm. **(E-E'') **Magnified view of the PPL2 cell cluster showing two PPL2 DA neurons (arrows) labeled with membrane tethered RFP. Two additional RFP-labeled neurons (arrowheads) do not express *TH *and correspond to neurons in which the *TH-gal4 *driver is ectopically activated. Scale bars: 10 μm. DA, dopaminergic; GFP, green fluorescent protein; L, instar larval stage; MARCM, mosaic analysis with a repressible cell marker; NB, neuroblast; *otd*, *orthodenticle*; PPL, protocerebral posterior lateral; RFP, red fluorescent protein; TH, tyrosine hydroxylase.

*otd *expression and *oc2 *enhancer activity in DA neurons of the larval and adult brain might establish an identity connection between the four larval DL2a DA neurons and the seven PPL2 DA neurons present in the adult brain. However, where did the additional three DA neurons present in the adult PPL2 cluster come from? A closer examination of L3 of the NB lineage that generates the DL2a DA neurons (Figure 5A-C) revealed that *otd *was expressed not only in the four TH-positive neurons, but also in three adjacent TH-negative cells (arrows in Figure 5A,B). These three cells might represent neurons born during larval development that acquire anti-TH immunoreactivity during pupal stages or, alternatively, they might be undifferentiated embryonic neurons that undergo phenotypic maturation only during pupal development. To distinguish between these two possibilities, twin-spot MARCM-labeled wild-type cell clones were induced in combination with the *TH-gal4 *driver during early L1 and adult brains were assayed for the presence of labeled DA neurons. In 13% of the analyzed adult brains (n = 30), one or two PPL2 DA neurons were labeled (Figure 5D-E), yet marker gene expression was not detected in three PPL2 DA neurons simultaneously. This result indicates that at least two PPL2 DA neurons in the adult brain are born during larval development. Conversely, the remaining five PPL2 DA neurons are likely to be of embryonic origin. Together, our data support the notion that the adult PPL2 cluster is derived from the larval DL2a cluster.

## Discussion

### Seven neuroblasts generate the larval central brain dopaminergic neurons

**Figure 6:**
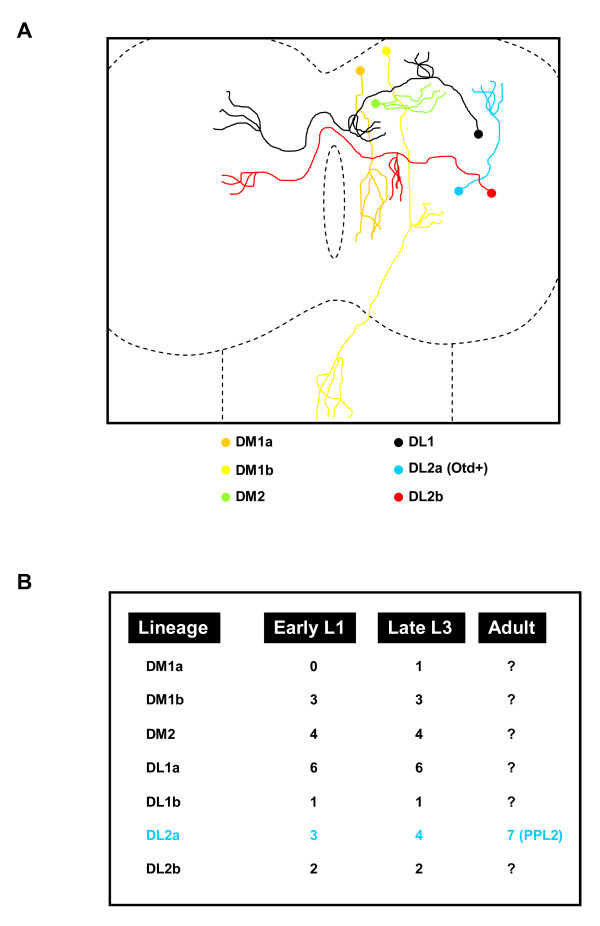
**Summary of the classification of dopaminergic neurons present in the larval central brain at L3, according to anatomical and lineage relationship criteria**. **(A) **A cartoon depicting a dorsal view of a L3 larva central brain showing cell body locations and neurite projection patterns of the different types of DA neurons. For simplicity, just one neuron per cluster and per brain hemisphere is shown. The cartoon does not pretend to precisely reproduce the innervation pattern of every type of DA neuron, but just gives a general description. **(B) **Table showing the distribution of DA neurons in different NB lineages at different times during development. The lineage harboring Otd+ DA neurons is highlighted in blue. DA, dopaminergic; DL, dorso lateral; DM, dorso medial; L, instar larval stage; NB, neuroblast; PPL, protocerebral posterior lateral.

The *Drosophila *larval central brain contains 21 DA neurons per hemisphere during L3, which express the cell type-specific marker gene *TH *[21,22]. Different methods have been proposed to classify and annotate these neurons according to anatomical criteria (position of the cell bodies within the cortex and/or projection pattern of the axonal tracts) [23-25]. In this paper, we have analyzed these neurons from a developmental point of view and classified them according to their lineage relationship (Figure 6). The MARCM technique is a powerful tool to study lineage progression and cellular pedigrees during *Drosophila *brain development [30]. It allows the labeling of progenitor cells and their offspring at different times during development, depending on the timing of a heat-shock-induced flippase-mediated mitotic recombination event [31]. Using this technique, we have shown that the larval central brain DA neurons are primary neurons born during early embryogenesis. However, when analyzing the lineage relationship among these neurons, two major problems were encountered. Firstly, implicit in the technique is the fact that a labeled NB clone is accompanied by a non-labeled twin clone (two post-mitotic cells derive from the first ganglion mother cell born just after the mitotic recombination event). The exclusion of two cells from the lineage analysis is negligible when larval lineages are analyzed (the average size of a standard larval lineage at L3 is 120 cells [32]). However, embryonic lineages are small (on average between 10 and 20 cells at the end of embryogenesis [3]) and the exclusion of two cells can be significant. Secondly, MARCM-labeled NB clones induced during early embryogenesis can only be visualized with a considerable delay after their generation (from L2 onwards) due to the persistence of the Gal80 repressor protein [30]. These two problems have recently been circumvented by the development of the twin-spot MARCM technique [33]. This technique not only allows the visualization of cell clones earlier in development but also differentially labels the NB clone and the twin clone; thus, the study of the entire NB lineage is now possible. Using this technique, we have analyzed the lineage relationship among the DA neurons present in the *Drosophila *central brain during larval development. We found that seven NB lineages generate the 21 DA neurons present in the larval central brain (Figures 2 and 6B; Additional file 1): DM1a (one DA neuron), DM1b (three DA neurons), DM2 (four DA neurons), DL1a (six DA neurons), DL1b (one DA neuron), DL2a (four DA neurons) and DL2b (two neurons). At large, the lineage analyses agree with the clustering of DA neurons according to anatomical criteria, supporting the general assumption that cell bodies arrangement and axonal projection patterns are reliable ways to classify neurons in *Drosophila*. Just in the case of the DL1 cell cluster was a discrepancy found. The cell bodies of the seven DL1 DA neurons are compactly arranged in a cell cluster that occupies medial-lateral positions in the L3 central brain and their neurites display similar projection patterns (Figure 1E). Yet, six DL1 DA neurons are clonally related (DL1a NB lineage; Figure 2C,C') and the remaining DL1 DA neuron (red asterisk in Figure 2C_2_) is generated by an additional NB (DL1b NB lineage). For future functional studies, it would be interesting to find molecular markers differentially labeling these two populations of DL1 DA neurons.

### Otd acts as a survival factor in DL2a dopaminergic neurons

Most studies involving the homeodomain transcription factor Otd in central nervous system development in *Drosophila *have dealt with its role in the specification and proliferation of progenitor cells during early neurogenesis [5,19], whereas a possible function in post-mitotic neurons has been largely overlooked. Our observation that *otd *is expressed in the DL2a DA neurons during larval development prompted us to investigate its role in the specification and/or survival of this DA cell cluster. According to anti-TH labeling, DL2a DA neurons mature mainly during early L1. Thus, null *otd *alleles, which are embryonic lethal, could not be used in our analysis. Therefore, we investigated the hypomorphic *otd *allele *oc*. We found that in *oc *mutant hemizygous larvae, *otd *expression in dorsolateral regions of the central brain was reduced and, as a consequence, only one of the four DL2a DA neurons showed anti-TH labeling during L3. The failure to detect three of the four DA neurons can be due to a defect in the regulation of *TH *expression or to the loss of DA neurons *per se*. Several lines of evidence support the latter hypothesis. Firstly, a general regulator of *TH *expression would be expected to be present in all or most of the central brain DA neurons; yet, *otd *expression during larval development is restricted to the DL2a DA cell cluster. Secondly, misexpression of *otd *in randomly induced cell clones in the central brain during larval development does not result in ectopic *TH*-expressing DA neurons (data not shown). Thirdly, labeling of DL2a DA neurons with the *oc2-gal4 *driver shows that reporter gene expression is also abolished in *oc *mutant hemizygous larvae during L3. The *oc2 *enhancer has been shown to be positively regulated by *otd *during ocelli development [28] and might not, therefore, be suitable to label DL2a DA neurons in an *otd*-independent way. However, a minimal version of this enhancer harboring the characterized Otd binding site (*oc7*) was active in the ocelli primordium [28], but did not show enhancer activity in DL2a DA neurons during larval development (data not shown). This indicates that the *oc2 *enhancer is differentially regulated in the ocelli primordium and in DA neurons during development and, hence, the *oc2-gal4 *driver may be used to label DL2a DA neurons in an *otd*-independent fashion.

Taken together, our observations support the hypothesis that *otd *expression is required for survival of DL2a DA neurons during larval development.

### DL2a dopaminergic neurons survive into adulthood and participate in the PPL2 dopaminergic cell cluster

The wild-type *Drosophila *adult brain is populated by about 200 DA neurons distributed in several bilaterally symmetric clusters [23,24,29]. The PPL2 cluster contains seven cells that express *otd *and five of them also show *oc2 *enhancer activity in young adult brains (Figure 4A_1_-A_4_). Similarly to the larval brain, *otd *expression in PPL2 DA neurons seems to be necessary for their survival, since neither anti-TH immunoreactivity nor transcriptional activity of the *oc2 *enhancer is detected in *oc *mutant adult brains (Figure 4B and data not shown). Moreover, the effects of targeted depletion of Otd in PPL2 DA neurons (loss of cell viability and/or *TH *expression; Figure 4C) can be rescued by the simultaneous expression of the anti-apoptotic gene *P35*, pointing out a role in cell survival as the main function of *otd *in PPL2 DA neurons. Altogether, the simplest interpretation for these results would be that *otd *expression labels homologous DA neuron populations in both the larval (DL2a cell cluster) and adult (PPL2 cell cluster) brains and, hence, both clusters contain the same DA neurons. The discrepancy in cell number between both clusters of DA neurons can be interpreted by analyzing the NB lineage responsible for the generation of the DL2a DA neurons. At L3, this lineage contains seven *otd *expressing cells, four of them are primary neurons that have already undergone maturation and express *TH*. The other three cells might represent immature secondary neurons that differentiate during pupal stages to give rise to the additional three DA neurons present in the adult PPL2 cluster. The distinction between early-differentiating (four cells) and late-differentiating (three cells) PPL2 DA neurons finds support in the targeted depletion of Otd in DA neurons by RNAi. Expression of an *otd*-specific RNAi construct in DA neurons (using the *TH-Gal4 *driver) has no effect on the larval brain (data not shown), but impairs the viability of four PPL2 DA neurons in the adult brain. Since these four cells differentiate during larval development, the RNAi machinery would have more time to completely deplete Otd than in the case of the late differentiating DA neurons. Further support for this interpretation also comes from the analysis in the adult brain of wild-type twin-spot MARCM cell clones induced during early L1. According to this analysis, at least two PPL2 DA neurons in the adult brain are secondary neurons, whereas the third DA neuron might represent an undifferentiated primary neuron that only matures during pupal development.

Recently, the expression of *Otx2*, an *otd *ortholog, in DA neurons in the mouse adult brain has also been reported [34]. It is selectively expressed in the central DA neurons of the ventral tegmental area, where it is cell autonomously required to antagonize identity features of the dorsal-lateral ventral tegmental area DA neurons [35]. Thus, contrary to *Drosophila*, depletion of Otx2 in these DA neurons does not induce cell death, but it changes neuron subtype identity. Interestingly, *otx2 *expression in these DA neurons has been associated with their reduced vulnerability to Parkinsonian neurodegeneration [35].

Finally, in *oc *mutant adult flies most of the protocerebral bridge, a neuropile structure that is part of the central complex, is also missing [20]. In several behavioral paradigms, these mutant flies walk slowly and show altered orientation behavior toward visual objects [36,37]. It has been recently proposed that the protocerebral bridge is an essential part of a visual targeting network that transmits directional clues to the motor output [37]. Thus, with regards to the data presented here, it would be interesting to analyze whether the lack of PPL2 DA neurons in *oc *mutant adult flies contributes to the behavioral phenotypes observed in these mutant flies.

## Conclusions

Using MARCM and twin-spot MARCM techniques together with anti-TH immunoreactivity, we have classified the 21 DA neurons present in the *Drosophila *larval central brain into seven clusters of clonally related DA neurons. The homeobox gene *otd *is specifically expressed in DA neurons belonging to one of these clusters (DL2a cluster); thus, *otd *expression differentially labels a subset of DA neurons. Furthermore, by taking advantage of an *otd *hypomorphic mutation and the *oc2-Gal4 *reporter line, we have established a cell lineage relationship between the larval DL2a and the adult PPL2 DA cell clusters. We also studied the role of *otd *in the survival and/or cell fate specification of these post-mitotic neurons. Contrary to mice, where *Otx2 *expression in DA neurons of the adult brain is necessary for neuron subtype identity, *otd *is required in the *Drosophila *larval and adult brain for survival of DL2a and PPL2 DA neurons. These findings suggest that *otd *acts as a post-mitotic selector gene whose differential expression among DA neurons might help to establish functional differences.

## Materials and methods

### Fly strains, clonal analysis and RNAi experiments

Flies were reared on standard medium at 25°C. The following transgene and reporter lines were used: UAS-*P35 *(Bloomington Drosophila Stock Center, Bloomington, Indiana, USA), UAS-*otd *(J Blanco, unpublished), *oc2-gal4 *[28], *TH-gal4 *[24]. Mutant alleles used in this study: *oc*^γa1^, *otd*^*YH13 *^[38].

Mitotic clones were generated and positively labeled (with membrane tethered CD8::GFP and CD2::RFP) according to the MARCM [30] and twin-spot MARCM [33] techniques. Unless indicated, recombination was induced 3 to 7 hours AEL by a one hour heat shock at 37°C and the larvae were dissected 21 hours later (early L1) or 110 hours later (L3). Genotypes of the analyzed larvae were as follows: *otd*^*YH13 *^MARCM clones, *w otd*^*YH13 *^*FRT19A*/*w hs-FLP tubP-GAL80*^*LL1 *^*FRT19A*; *tubP-GAL4 UAS-mCD8::GFP*^*LL5*^/*+*;

wild-type MARCM clones, *y w hs-FLP/+*; *FRT82*/*FRT82 tubP-GAL80*^*LL10*^; *tubP-GAL4*^*LL7 *^(or *TH-GAL4*) *UAS-mCD8::GFP*^*LL6*^/*UAS-mCD8::GFP*^*LL6*^; wild-type twin-spot MARCM clones, *y w hs-FLP/+*; *FRT40A UAS-mCD8::GFP UAS-CD2-Mir*/*FRT40A UAS-rCD2::RFP UAS-GFP-Mir*; *tubP-GAL4*^*LL7 *^(or *TH-GAL4*)/*+*.

Depletion of Otd by RNAi was carried out by targeted expression of an *otd*-specific RNAi construct (VDRC-105764) in DA neurons using the *TH-gal4 *driver. To increase knockdown efficiency, the experiment was done at 29°C in *otd*^*YH13 *^heterozygous flies.

### Immunohistochemistry

Antibody staining on brains was performed as previously described [39]. Primary antibodies were as follows: rabbit anti-Otd (1:250) [40], mouse anti-TH (1:100; Chemicon, Millipore AG, Temecula, California, USA), rabbit anti-TH (1:250) [41], rabbit anti-RFP (1:100; Abcam, Cambridge, UK). Secondary antibodies were Alexa488-, Alexa568- and Alexa647-conjugated antibodies generated in goat (1:200; Molecular Probes, Invitrogen, Paisley, Renfrewshire, UK). Fluorescent images were captured with an Olympus FV1000 confocal laser scanning microscope and analyzed in ImageJ [42]. Unless otherwise indicated, pictures correspond to single optical sections (1 μm thick). Figures were assembled using Adobe Illustrator and Photoshop.

## Abbreviations

AEL: after egg laying; DA, dopaminergic; DL: dorso lateral; DM: dorso medial; FRT: flippase recognition target; GFP: green fluorescent protein; L: instar larval stage; MARCM: mosaic analysis with a repressible cell marker; NB: neuroblast; *oc*: *ocelliless*; *otd*: *orthodenticle*; PPL: protocerebral posterior lateral; RFP: red fluorescent protein; RNAi: RNA interference; TH: tyrosine hydroxylase.

## Competing interests

The authors declare that they have no competing interests.

## Authors' contributions

JB and RP carried out all the experiments. JB, MW and GU conceptualized the project. JB and GU wrote the manuscript. All authors read and approved the final manuscript.

## Supplementary Material

Additional file 1 **Supplementary Figure 1 - MARCM lineage analysis of late differentiating dopaminergic neurons in the larval central brain at L3**. MARCM-labeled wild-type NB clones were induced during early embryogenesis (3 to 7 h AEL) and analyzed at L3. To circumvent the down-regulation of the *tubulin *promoter, an additional copy of the *UAS-CD8::GFP *transgene was included in the genotype of the analyzed larvae. **(A-C) **The DM1a DA neuron belongs to a NB lineage independent of the DM1b DA cell lineage. **(D,E) **The late differentiating DL2 DA neuron belongs to the DL2a DA cell lineage. Scale bars: 10 μm. All panels correspond to Z projections of individual confocal optical sections. AEL, after egg laying; DA, dopaminergic; DL, dorso lateral; DM, dorso medial; GFP, green fluorescent protein; MARCM, mosaic analysis with a repressible cell marker; NB, neuroblast. Click here for file
