# Supplementary figures and images for: Orthodenticle is necessary for survival of a cluster of clonally related dopaminergic neurons in the Drosophila larval and adult brain
Source: Neural Dev. 2011 Oct 14;6:34. doi: 10.1186/1749-8104-6-34 (PMC3206411; doi:10.1186/1749-8104-6-34)

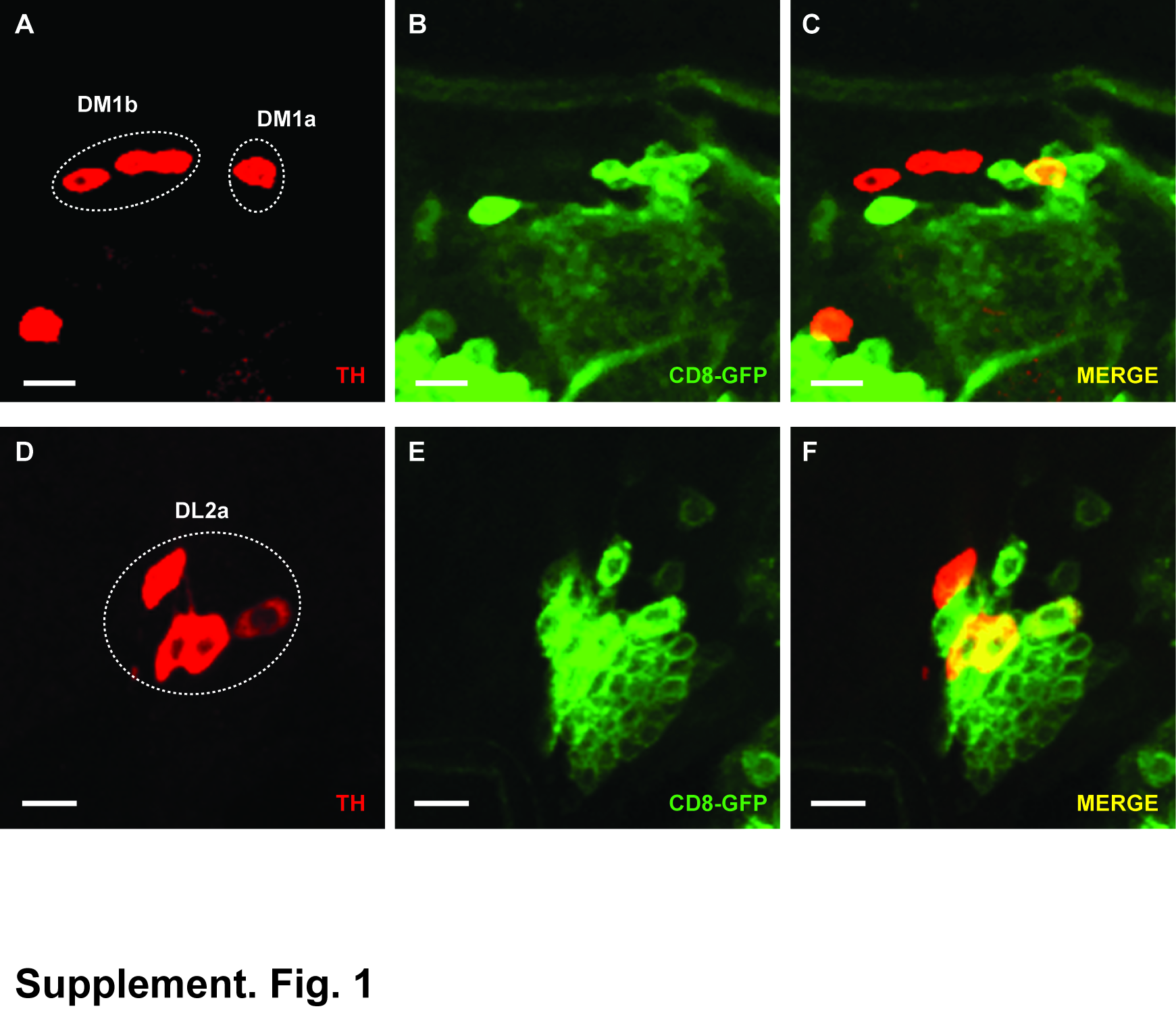

Supplement: Additional file 1 — Supplementary Figure 1 - MARCM lineage analysis of late differentiating dopaminergic neurons in the larval central brain at L3. MARCM-labeled wild-type NB clones were induced during early embryogenesis (3 to 7 h AEL) and analyzed at L3. To circumvent the down-regulation of the tubulin promoter, an additional copy of the UAS-CD8::GFP transgene was included in the genotype of the analyzed larvae. (A-C) The DM1a DA neuron belongs to a NB lineage independent of the DM1b DA cell lineage. (D,E) The late differentiating DL2 DA neuron belongs to the DL2a DA cell lineage. Scale bars: 10 μm. All panels correspond to Z projections of individual confocal optical sections. AEL, after egg laying; DA, dopaminergic; DL, dorso lateral; DM, dorso medial; GFP, green fluorescent protein; MARCM, mosaic analysis with a repressible cell marker; NB, neuroblast. [file 1749-8104-6-34-S1.TIFF]
